# A Longitudinal Dyadic Study of Six Leisure Activities in Swedish Couples During the Transition to Parenthood

**DOI:** 10.1007/s11199-023-01351-3

**Published:** 2023-02-24

**Authors:** Lucy R. Zheng, Elin Naurin, Elias Markstedt, Petrus Olander, Helen Elden, Karolina Linden

**Affiliations:** 1 Department of Political Science, University of Gothenburg, Sprängkullsgatan 19, 41123 Göteborg, Sweden; 2 Society, Opinion and Media Institute, University of Gothenburg, Seminariegatan 1B, 413 13 Göteborg, Sweden; 3 Institute of Health and Care Sciences, Sahlgrenska Academy, University of Gothenburg, Box 457, 405 30 Göteborg, Sweden; 4 Region Västra Götaland, Department of Obstetrics and Gynecology, Sahlgrenska University Hospital, Diagnosvägen 15, 416 50 Göteborg, Sweden

**Keywords:** Leisure, Activities, Longitudinal, Transition to parenthood, Couples, Sweden

## Abstract

Parents are not only caregivers to their children; they also have leisure routines that can impact their own well-being. However, little is known about how leisure activities change within the context of a couple during the transition to parenthood. This study uses latent growth curve models and data from the Swedish Pregnancy Panel to examine how often 918 first-time, heterosexual couples participated in six leisure activities from around pregnancy week 19 to one year postpartum. Compared to fathers, mothers less frequently exercised and listened to news, and more frequently read newspapers, spent time for themselves, and spent time with friends. Over time, mothers increased their frequency of praying to God and decreased spending time for themselves and with friends. Fathers decreased frequency of exercise. Within couples, there was a positive correlation between mothers’ and fathers’ frequency of engaging in leisure activities, although most changes over time were not associated. Our finding that two individuals within a couple may change their leisure activities independently of each other during the transition to parenthood can help healthcare professionals and researchers prepare expectant couples for upcoming changes (or lack thereof) and promote parent well-being. Our findings also highlight the possibility that in contexts with more state support for families, parenthood may not exacerbate gender gaps in leisure.

## Introduction

For expectant couples, the transition to parenthood is an exciting phase of life. A process that typically begins with pregnancy, the transition to parenthood is a complex interplay of expectations and responsibilities across personal, interpersonal, and contextual domains (Nelson et al., 2014). Mothers and fathers build skills, resources, relationships, and behaviors to meet the new demands of raising another human (Deave et al., 2008; Rowe et al., 2013). However, being a good parent and caregiver also requires that parents are physically and mentally healthy (Foster et al., 2010; Nomaguchi & Milkie, 2020).

Parental well-being is a combination of parenting-related and individual well-being (Ketner et al., 2019). While the former refers to a sense of achievement related to their role as a parent, the latter refers to the extent to which a parent experiences happiness with one’s own life and with oneself. One aspect of daily life that may contribute to this individual well-being is leisure activities, activities in which individuals participate voluntarily and that are not directly related to paid work or unpaid work (childcare, housework) (Craig & Mullan, 2013). Since leisure is linked to positive emotions (Brajša-Žganec et al., 2011; Kim et al., 2018), effective coping strategies (Pöllänen, 2015; Qian et al., 2013; Weng & Chiang, 2014), and fewer mental health symptoms and improved parenting behaviors (Brenning & Soenens, 2017), it is important for society to understand how these influences of well-being change during the transition to parenthood, a highly exciting yet stressful period. However, beyond just knowing how parenthood might influence leisure, the field can benefit from a clearer look into the dyadic interplay in leisure between mothers and fathers (Mickelson & Biehle, 2017). When a first child is born, couples may experience changes in their roles and have to work together to balance caring for an infant and other responsibilities and tasks (Doss & Rhoades, 2017). Since time is limited, each partner’s leisure might affect the leisure time available to the other partner (Umberson et al., 2018), making the couple-dynamic central to our understanding of leisure changes.

This current study uses longitudinal data to investigate the trajectories of six leisure activities for first-time expectant mothers and fathers from the second trimester to one year postpartum. A sample of 918 heterosexual couples living in Sweden were recruited during pregnancy and surveyed twice during the pregnancy and twice after childbirth. The longitudinal dyadic design allows for latent growth curve models that clarify (1) differences in frequency of leisure activities between the mother and the father, (2) how frequency of leisure activities changes for each parent, as well as (3) how the couple influences each other’s frequency of leisure activities over time.

### Well-Being During the Transition to Parenthood

*“Parents are not merely caregivers to their children.”* (Ketner et al., 2019, p. 275).

Aside from being a caregiver to a child, a parent has individual wishes and problems (Petterson et al., 2016). Self-determination theory states that positive human development is characterized by not only being aware of one’s needs, values, and goals, but also the ability and autonomy in reaching these aspirations (Deci & Ryan, 2012). Given that maintaining certain activities (creative arts, music, physical activity, social interactions) during pregnancy and parenthood is linked to well-being (Chang et al., 2015; Demecs et al., 2011; Khoury et al., 2021; Liu et al., 2019), it is important to better understand how activities with the potential to buffer against stresses change during the early transition to parenthood.

Research has found that beyond the happiness that a child brings (Aassve et al., 2012; Nelson et al., 2013), the transition to parenthood brings with it a decrease in how adults rate their life (Deaton & Stone, 2014), fewer friends and acquaintances and less support (Kalmijn, 2012), and an increase in mental health problems (O’Hara & Wisner, 2014). Accounts of new parenthood in both mothers and fathers report “role strain” and “role conflict” around the loss of their former life and activities (Bowyer et al., 2022; Darwin et al., 2017; Shepherd-Banigan et al., 2016; Zubair et al., 2021). Since well-being is not only tied to parent mental health, but also supportive, positive parenting behaviors (Sameroff, 2009), it is important to understand the factors that support parent well-being.

Research on leisure activities across age groups and the lifespan finds that men generally spend more time on leisure compared to women (Anxo et al., 2011; García Román & Gracia, 2022; Giurge et al., 2021). This gap continues to exist after becoming parents (Beblo, 2001; Bianchi & Milkie, 2010; Craig & Mullan, 2013; Latshaw & Hale, 2016; Mattingly & Bianchi, 2003). However, as noted by Anxo and colleagues, early parenthood sees a shrinking in the gender gap, almost as if “inequalities in leisure time between parents would be considered unfair” (2011, p. 185). Since this transition to parenthood can spark housework, child care, and leisure routines that can last for years and make enduring impacts on health and well-being (Alderdice & Lynn, 2009; Baxter et al., 2008; Hansson & Ahlborg, 2016; Medeiros & Piccinini, 2019), it is crucial to study how leisure changes during the transition to parenthood, a period of physiological, psychological, and social change (Bäckström et al., 2018; Klobučar, 2016).

### Defining Leisure

Leisure activities have been defined in many ways: activities that provide pleasure and happiness in life (Aristotle, 1998); activities that create and maintain life quality (Leung & Lee, 2005); activities that bring a state of mind of “pleasure or contentment” (Erkip, 2009). Many leisure articles do not explicitly define leisure but rather the activities themselves, such as vacation, socializing with friends, watching television, or physical activity/sports (Brajša-Žganec et al., 2011; Kamp Dush et al., 2018; Mirehie & Gibson, 2020; Schmiedeberg & Schröder, 2017; Wiese et al., 2018). Other articles refer to activities as leisure if they have positive benefits for health and well-being (Moses et al., 2016; Qian et al., 2014). The consensus for an actual definition of leisure activities, however, is that they are activities that individuals take part in during their free time (Dattilo & Kleiber, 1993; Demirbaş, 2020; Pressman et al., 2009; Stebbins, 2005) and excludes activities related to paid work and unpaid care work (domestic labor and childcare) (Craig & Mullan, 2013).

Leisure in this paper refers to such free-time activities. We do not exclude any activity within the confines of that broad terminology (e.g., excluding an activity based on enjoyment) to avoid contributing to biases as to what is enjoyable and what is not. For instance, exercise may be perceived as an obligation for certain individuals but a mainly enjoyable activity for others. Along the same lines, some may find reading, listening to, or following the news (or political engagement generally) enjoyable, while others consider it a rather tedious type of responsibility that they need to spend their free time on. Thus, in our study, we take a broad conceptualization of leisure. Following an interpretation common among researchers, we refer to leisure activities as activities that are not paid work or unpaid care-related activities done for someone else.

### Leisure Activities and Couple Dynamics

Previous studies during the transition to parenthood found that parents report a lower frequency of physical activity and engagement in politics (Abbasi & van den Akker, 2015; Naurin et al., 2022; Sjögren Forss & Stjernberg, 2019). Beyond a general decrease in leisure activities, there may also be gender differences during this transition. Previous studies have found that fathers tend to have more leisure time as a parent (Beblo, 2001; Craig & Mullan, 2013; Giurge et al., 2021; Mattingly & Bianchi, 2003; Office for National Statistics, 2018), although less is known about leisure during this life transition where responsibilities and priorities shift, and the dyadic interplay in leisure between mothers and fathers (Mickelson & Biehle, 2017). In a 2021 study with data from over 31,000 participants from the United States, Canada, Denmark, Brazil, and Spain, researchers found that while women in the overall sample participated in less leisure, gender differences were driven by the parents in the sample (Giurge et al., 2021). In a study with nationally representative samples from Australia, United States, France, Italy, and Denmark, the quantity and quality of leisure time favor fathers (Craig & Mullan, 2013). In the United States, studies have found that fathers consistently take more leisure time than mothers (Beblo, 2001), each additional child reduces the amount of leisure time more for mothers than for fathers (Mattingly & Bianchi, 2003), and the presence of small children reduces leisure time more for women than men (Latshaw & Hale, 2016).

However, mothers and fathers do not exist within a vacuum; rather, their activity levels depend upon their partners. When a first child is born, couples may experience changes in their roles and have to work together to balance caring for an infant and other responsibilities and tasks (Doss & Rhoades, 2017). Since time is limited, each partner’s leisure might affect the leisure time available to the other partner (Umberson et al., 2018), making the couple-dynamic central to our understanding of leisure changes. In the only study on leisure time of expecting parents, researchers found that among the 182 primarily highly-educated, White, dual-earner U.S. couples, fathers enjoyed more leisure time and engaged in more leisure while mothers performed more childcare and routine housework (Kamp Dush et al., 2018). While insightful, this study does not show how individual activities during this time shift and how the couple’s activities are associated over time. In addition, the study utilized a small sample in the United States, a context with limited state support and more conservative gender norms compared with other Western contexts (Nadler & Lowery, 2018).

### Outstanding Questions in the Literature

There are three main shortcomings in previous work. Much of the leisure literature is based on samples from the United States and the United Kingdom (Claxton & Perry-Jenkins, 2008; Kamp Dush et al., 2018; Mattingly & Bianchi, 2003; Offer, 2016; Office for National Statistics, 2018; Sayer, 2005; Wang, 2013), where state support for families and childcare is weak (Chzhen et al., 2019; Fleming, 2019; Gromada & Richardson, 2021). Social institutions such as laws and guidelines shape and are shaped by the beliefs of that particular society and context (Heymann et al., 2019; Jütting et al., 2008; Kahan, 2000). Thus, findings from studies conducted in the United States and the United Kingdom may only be generalizable to contexts with less state support for families, which also coincides with gender inequality in parenting (Ferrant et al., 2014). Since context is important for understanding behavior, leisure research will benefit from studies conducted in a context with more state support for families and more gender-equal norms. Sweden is ranked first in the EU on the Gender Equality Index (European Institute for Gender Equality (EIGE), 2021) and seventh worldwide (United Nations Development Programme, 2020), with strong support for families (e.g., childcare, paid parental leave) (Duvander et al., 2005; Oláh & Bernhardt, 2008). These policies may encourage men and women to share similar levels of leisure time in the same way men and women more equally share parental labor (Boye & Evertsson, 2014; Duvander et al., 2017). In a 2017 study with data from 23 countries, researchers find that the gender gap in leisure quality is smaller when gender norms are more egalitarian (Yerkes, Roeters, et al., 2020).

Second, conceptualizing the gender differences (and similarities) in leisure activities requires understanding of not just total leisure time, but individual leisure activities as well. Studies on leisure of couples combine all leisure into one composite variable (Bittman & Wajcman, 2000; Craig & Mullan, 2013; Giurge et al., 2021; Offer, 2016; Thrane, 2000; Yerkes, André, et al., 2020; Yerkes, Roeters, et al., 2020). However, given our broad conceptualization of leisure, which does not take into account subjective enjoyment, examining distinct leisure activities, which differ in physical and psychological involvement (Li et al., 2021), can provide an alternative view to leisure routines and change during this time. Only one cross-sectional report from the Pew Research Center (following United States samples) compares individual leisure activities for parents, and finds that fathers spend more time playing sports or exercising than do mothers, while mothers spend more of their leisure time in social activities such as attending or hosting parties (Wang, 2013). However, the study investigated leisure for mothers and fathers in general (not couples) and did not follow them over time.

Third, while the field knows about leisure during parenting, little has been done to investigate a couple’s leisure activity trajectories during the transition to parenthood. Given the importance of leisure activities, it is important for research to understand the dynamics of leisure within a couple (Mickelson & Biehle, 2017) particularly during this time of change. To our knowledge, no previous research has explored the dyadic relationship of individual leisure activities between couples starting in pregnancy and throughout the transition to parenthood.

### Purpose of the Present Study

The present study addresses three main research questions.

1. How does frequency of six leisure activities differ between expectant mothers and fathers around pregnancy week 19?
2. Does the frequency of activities change over the course of pregnancy and early parenthood?
3. Are frequency and change of activities associated between mothers and fathers?

## Method

### Participants

In this study, we use data from the Swedish Pregnancy Panel (Naurin et al., 2021), which is a longitudinal survey study of pregnant women and their partners, combined with data from the Swedish Pregnancy Register, which is a national quality register (Stephansson et al., 2018). The Swedish Ethical Review Authority approved the Swedish Pregnancy Panel on April 15, 2019 (Dnr: 1061-18) prior to data collection. We use panel data from first-time expectant heterosexual couples around pregnancy week 19, pregnancy week 36, 2 months after childbirth (postpartum), and 1 year after childbirth (postpartum). Couples from same-sex relationships and those with experience of previous births were excluded. Participants were sent the first survey between pregnancy week 12 and 19, but the median response was during pregnancy week 19. The timing of the first survey, which was automatically sent upon being recruited, depends on when they were recruited.

Recruitment occurred in the ultrasound waiting area of a tertiary care hospital in Sweden, the largest birthing hospital in the country, with around 10,000 births per year (Graviditetsregistret, 2020). Almost all women in Sweden undergo these routine ultrasound scans, due to the free antenatal care offered to all pregnant women. Participants were recruited as they were waiting for the scheduled first or second trimester routine ultrasound screening. 96% of all the routine second-trimester ultrasound and 55% of all first trimester ultrasounds in the studied city area were performed at the hospital where recruitment took place. Thus, the current sample was recruited from a general pregnant population within the city area.

Participants were recruited in face-to-face approaches, signed paper consent forms, and filled in a self-administered profile questionnaire on tablets. Questionnaires in subsequent waves were sent online to participants. If participants did not respond, email reminders were sent after one and two weeks and telephone reminders were given after three weeks. To incentivize participation, participants were given a 100 SEK gift card after completion of the survey at pregnancy week 22–24. The questionnaires could be taken in Swedish, English, Arabic, or Somali, although 92.9–95.3% took it in Swedish across all three questionnaires. Data collection ranged from September 2019 to October 2021.

**Table 1:** Characteristics of First-Time, Heterosexual Couples Around Pregnancy Week 19, Pregnancy Week 36, 2 Months Postpartum, and 1 Year Postpartum

	Percentage of Sample (%)							
	Pregnancy Week 19 *N* = 918 Couples		Pregnancy Week 36 *N* = 657 Couples		2 Months Postpartum *N* = 606 Couples		1 Year Postpartum *N* = 453 Couples	
	Mothers	Fathers	Mothers	Fathers	Mothers	Fathers	Mothers	Fathers
**Age**								
21–25	4.6	1.9	3.5	0.9	2.9	0.8	2.9	0.9
26–30	27.3	19.6	26.7	18.1	26.1	17.0	25.9	15.4
31–35	49.4	45.3	49.9	45.4	50.9	43.8	52.1	45.2
36–40	14.9	23.2	16.0	24.7	16.1	26.3	15.2	27.1
40+	3.8	10.0	3.9	11.0	4.0	12.0	3.9	11.4
**Citizenship**								
Swedish citizen	84.3	83.2	85.3	84.1	86.2	84.8	85.6	83.7
Citizen of another country	10.5	9.5	9.3	9.2	8.7	8.6	8.6	9.3
Dual citizen (Sweden and another country)	5.2	7.2	5.4	6.7	5.1	6.6	5.8	7.0
Marital status*								
Married	36.0	39.0	35.3	40.8	35.1	41.2	35.5	40.1
Committed relationship	64.0	62.2	65.0	60.2	65.5	60.2	65.2	61.8
**Education**								
< 3 years of high school	2.1	2.3	2.0	1.2	1.7	1.0	1.2	0.7
High school, ≥ 3 years	13.7	18.5	12.4	16.7	12.4	15.2	11.5	11.6
Post-high school, non-tertiary < 3 years	7.6	10.1	7.3	11.2	7.5	11.3	7.8	9.9
Post-high school, non-tertiary ≥ 3 years	2.9	1.8	2.7	1.6	2.9	1.6	2.9	1.4
Tertiary education, < 3 years	6.1	8.1	5.9	7.5	5.2	7.9	6.0	7.3
Tertiary education, ≥ 3 years	62.0	53.6	65.3	55.8	66.5	58.0	66.7	63.8
Postgraduate education	4.6	5.6	4.4	5.8	3.7	5.0	3.9	5.0
**Annual household income**								
≤ SEK 300,000	6.7	4.7	6.6	4.5	6.2	3.0	6.1	2.4
SEK 301,000–400,000	4.7	3.5	4.5	3.4	4.2	3.6	3.8	3.0
SEK 401,000–500,000	6.1	6.9	6.4	5.7	6.4	5.1	6.5	4.9
SEK 501,000–600,000	7.3	7.6	6.6	7.6	7.4	7.0	7.0	7.4
SEK 601,000–700,000	14.3	12.0	13.6	12.0	13.4	10.8	12.6	10.6
SEK 701,000–800,000	17.9	20.6	18.7	20.6	20.1	20.7	19.6	20.4
SEK 801,000–900,000	18.4	18.1	18.5	17.9	18.0	18.6	20.3	18.0
SEK 901,000–1,000,000	13.3	12.7	14.0	13.2	12.4	14.0	13.6	14.8
> SEK 1,000,000	11.2	14.0	11.2	15.1	11.9	16.1	10.5	17.5

*Note*. *Participants had the option to check all options that applied; hence, percentages may not add up to 100%.

### Measures

We take a structural rather than subjective approach to conceptualizing leisure. In other words, we emphasize how leisure is structured by activity rather than measure the total amount of leisure time without specifying what constitutes leisure (Kelly & Godbey, 1992; Kleiber et al., 2011; Newman et al., 2014). Leisure time spent was measured by the question “In the last three months, how often have you done the following?”. We analyze answers regarding six leisure activities, ordered here according to how often participants report doing them: “Listened to the news on the radio/podcast/TV”, “Exercised/gone to the gym/done sports”, “Read a newspaper or news tabloid”, “Spent time just for myself”, “Spent time with friends”, “Prayed to God”. These activities were included in a battery of items that also included other activities that were not leisure. Participants responded to each activity on a 7-point scale, with the response options 1 “*Never*”, 2 “*Sometime during the last 3 months*”, 3 “*About once a month*”, 4 “*Several times a month*”, 5 “*About once a week*”, 6 “*Several times a week*”, and 7 “*Every day*”. Participants did not see any numbers attached to the response labels. Our use of the Likert scale is similar to previous articles on leisure that took the structural approach (Kim et al., 2016; Paggi et al., 2016; Schmiedeberg & Schröder, 2017; Sjögren Forss & Stjernberg, 2019).

We control for two factors related to the context in which leisure activities take place. These factors include economic capital (household income) and human capital (education). Higher income and education provide access to social networks and resources (Bianchi & Vohs, 2016; Heritage et al., 2008; Mirowsky & Ross, 2017; Nie et al., 1996) and support the development of gender egalitarian attitudes (Canzio, 2020) that could encourage a balanced distribution of leisure. Data on whether it was the mother’s first time giving birth was obtained from the register.

### Statistical Analysis

Analyses were conducted using R version 4.0.3 and *lavaan* package version 0.6.9. We used a robust maximum likelihood estimator (MLR) to account for non-normal distributions of observed variables and full information maximum likelihood procedure to account for missing data.

Each activity was modeled individually as a linear latent growth curve model (LGCM; Preacher et al., 2008), using the dyadic data structure that differentiated between mothers and fathers and controlling for household income and educational attainment. Model fit indices are shown in Table 2. The linear model for “spent time just for myself” had poor fit (CFI = 0.67, TLI = 0.58, RMSEA = 0.13), suggesting that individual trajectories did not consistently conform to a linear trend. Thus, we tested alternative models, including an LCGM with quadratic parameters and a piecewise latent growth curve model (PLGCM), a model that breaks up the growth trend into separate segments and allows pieces of the trajectory to have their own slope estimations (Kohli & Harring, 2013). However, the lack of significant improvement in fit and desire for model parsimony led us to keep the linear model. No other model modifications were made.

**Table 2:** Model Fit Indices for Latent Growth Curves - Mothers’ and Fathers’ Intercepts and Slopes of Leisure Activities

	Listen to news	Exercised/ Gone to the Gym	Read Newspaper	Spent Time for Myself	Spent Time with Friends	Prayed to God	
**CFI**	0.99	0.88	0.97	0.67/0.76/0.72	0.90	0.97	
**TLI**	0.98	0.85	0.97	0.58/0.41/0.57	0.88	0.96	
**RMSEA**	0.04	0.09	0.05	0.13/0.15/0.13	0.09	0.10	
**90% CI RMSEA**	0.00-0.06	0.07 − 0.01	0.03–0.07	0.11–0.15/0.13–0.18/0.11–0.15	0.07–0.11	0.08–0.12	
**SRMR**	0.04	0.06	0.04	0.09/0.08/0.08	0.08	0.04	
**χ2**	48.94	108.71	63.20	180.25/131.76/152.11	110.59	127.29	
***df***	37	35	38	35/18/29	37	37	
***p***	0.09	0.00	0.01	0.00/0.00/0.00	0.00	0.00	

*Note.* Values in the table indicate the fit indices for the linear model with controls and constraints (degrees of freedom differ based on constraints). χ^2^ = scaled chi-square; df = degrees of freedom; CFI = comparative fit index; TLI = Tucker-Lewis Index; RMSEA = root-mean-square error of approximation; CI = confidence interval; SRMR = standardized root mean square residual. CFI/TLI > 0.95, RMSEA < 0.06, SRMR < 0.08 are considered good fit. For “spent time for myself”, values left of the slash (/) reflect fit indices from the linear growth model, values in the middle reflect indices from the quadratic growth model, and values to the right reflect fit indices from the piecewise growth model. The lack of significant improvement and desire for parsimony in model creation led us to keep the linear model (left of the slash).

Constraints were used to determine whether intercepts and slopes were significantly different for expectant mothers and fathers. Intercepts are estimates of the activity level around pregnancy week 19 and slopes are the estimates of activity change from pregnancy week 19 to 1 year postpartum. Covariances from the LGCM were used to determine associations between mothers’ and fathers’ intercepts and slopes. Significance levels were set at *p* < .05.

## Results

### Preliminary Analysis

At pregnancy week 19, pregnancy week 36, 2 months postpartum, and 1 year postpartum, our sample consisted of 918, 657, 606, and 453 couples, respectively. While FIML does account for values that are missing for each individual, dyadic analyses require data from both couple members, Thus, we are able to address missingness of values from couples where both members responded to the survey, even if they didn’t respond to the specified leisure questions. However, when one couple member did not respond, the couple was dropped from the analyses. At the level of the dyad, fathers were responding on average at 65% of the mother’s rate.

Attrition analyses of study variables found no significant differences (*p* < .05) between those who dropped out and those who responded at 1 year postpartum on frequency of exercise, time with friends, reading newspaper or news tabloids, listening to news around pregnancy week 19, and income. However, participants who dropped out spent less time for themselves and more time praying to God around pregnancy week 19. In addition, participants who responded at 1 year after childbirth were older and had higher educational attainment than those who dropped out.

Most of the first-time expectant mothers and fathers in the Swedish Pregnancy Panel were Swedish citizens and had at least three years of tertiary education. Almost all the participants were either married or cohabiting (98.4% around pregnancy week 19, 98.8% at 1 year postpartum) and about half the sample was between the ages of 31–35 at the beginning of the study. Three-quarters of the sample had a household income above SEK 600,000 annually before taxes.

Intercepts and slopes from the latent growth curve models are presented in Table 3. Variances and associations between mothers and fathers (covariances) are shown in Table 4.

**Table 3:** Intercepts and slopes of leisure activities, by first-time mothers and fathers

	Mother		Father	
	Intercept (SE) ^a^	Slope (SE)	Intercept (SE) ^a^	Slope (SE)
Listened to the news	4.11 (0.65)*	-0.20 (0.14)	5.10 (0.63)*	-0.20 (0.14)
Exercised/gone to the gym/sports	3.94 (0.65)*	0.10 (0.20)	4.43 (0.65)*	-0.49 (0.20)*
Read a newspaper or news tabloid	2.72 (0.86)*	0.07 (0.17)	2.07 (0.87)*	0.07 (0.17)
Spent time just for myself	3.58 (0.50)*	-0.46 (0.19)*	3.15 (0.57)*	0.03 (0.17)
Spent time with friends	3.33 (0.46)*	-0.27 (0.14)*	2.92 (0.44)*	-0.14 (0.13)
Prayed to God	1.25 (0.45)*	0.17 (0.07)*	1.25 (0.45)*	-0.07 (0.08)

*Note.* Intercepts and slopes were obtained from latent growth curve models. Parameters with same numbers across mother and father indicate that mother and father were not significantly different (constrained model). Parameters with different numbers across mother and father indicate that mother and father were significantly different (non-constrained model). Models were adjusted for household income and education.

^a^ Intercepts correspond to actual labels within quotes that participants saw in response to the question “In the last three months, how often have you done the following:”: (1) *Never* (2) *Sometime during the last 3 months* (3) *About once a month* (4) *Several times a month* (5) *About once a week* (6) *Several times a week* (7) *Every day*.

*p < .05.

**Table 4:** Covariances and variances between first-time mothers’ and fathers’ intercepts and slopes of leisure activities

		Mother Leisure Activity		Father Leisure Activity	
		Intercept (I)	Slope (S)	Intercept (I)	Slope (S)
**Mother leisure activity**					
Listened to the news	I	2.02 (0.19)*	-0.16 (0.05)*	0.83 (0.19)*	-0.11 (0.05)*
	S	-	0.09 (0.02)*	0.00 (0.05)	0.03 (0.01)*
Exercised/gone to the gym	I	1.39 (0.24)*	0.02 (0.06)	0.71 (0.18)*	-0.08 (0.05)
	S	-	0.02 (0.03)	0.01 (0.05)	**0.02 (0.02)**
Read a newspaper/news tabloid	I	3.60 (0.32)*	-0.18 (0.07)*	1.57 (0.32)*	-0.06 (0.08)
	S	-	0.08 (0.02)*	-0.05 (0.08)	**0.02 (0.02)**
Spent time for myself	I	0.54 (0.14)*	0.02 (0.04)	**-0.03 (0.12)**	0.03 (0.04)
	S		0.02 (0.01)	0.11 (0.04)*	**-0.02 (0.01)**
Spent time with friends	I	0.72 (0.09)*	0.00 (0.02)	0.37 (0.09)*	-0.02 (0.02)
	S	-	0.01 (0.01)	0.02 (0.02)	**0.00 (0.01)**
Prayed to God	I	1.58 (0.12)*	-0.02 (0.01)	0.70 (0.12)*	-0.04 (0.02)*
	S	-	0.00 (0.00)	-0.02 (0.01)	**0.00 (0.00)**
**Father leisure activity**					
Listened to the news	I	-	-	2.02 (0.19)*	-0.14 (0.05)*
	S	-	-	-	0.03 (0.02)
Exercised/gone to the gym	I	-	-	1.82 (0.24)*	-0.15 (0.06)*
	S	-	-	-	0.09 (0.02)*
Read a newspaper/news tabloid	I	-	-	3.60 (0.32)*	-0.16 (0.07)*
	S	-	-	-	0.08 (0.02)*
Spent time for myself	I	-	-	1.17 (0.18)	-0.02 (0.04)
	S	-	-	-	0.02 (0.01)
Spent time with friends	I	-	-	0.72 (0.09)*	0.03 (0.02)
	S	-	-	-	0.01 (0.01)
Prayed to God	I	-	-	1.58 (0.12)*	-0.07 (0.02)*
	S	-	-	-	0.03 (0.00)*

*Note.* Parameters are covariances and variances derived from latent growth curve models. Bolded parameters are associations of the same parameters (intercept and slope) across expectant mothers and fathers. Associations with same parameters indicate variances. Associations between different parameters indicate covariances. Models were adjusted for household income and education. Participants responded to “In the last three months, how often have you done the following:”.

*p < .05.

### Research Question 1: How Does Frequency of Six Leisure Activities Differ Between Expectant Mothers and Fathers Around Pregnancy Week 19?

At the start of our measurement around pregnancy week 19, expectant mothers were less likely than fathers to listen to the news and exercise, but more likely to read a newspaper or news tabloid, spend time for herself, and spend time with friends. While expectant fathers listened to the news about once a week (B_I_ = 5.10, SE = 0.63), mothers tended to listen less, several times a month (B_I_ = 4.11, SE = 0.65). Fathers also reported exercising more (B_I_ = 4.43, SE = 0.65) compared to mothers (B_I_ = 3.94, SE = 0.65), although the value label was “several times a month” for both.

On the other hand, mothers read newspapers or news tabloids about once a month (B_I_ = 2.72, SE = 0.86), whereas fathers read them less, sometime during the last few months (B_I_ = 2.07, SE = 0.87). Mothers reported significantly higher frequency in spending time just for themselves (B_I_ = 3.58, SE = 0.50) and with friends (B_I_ = 3.33, SE = 0.46), compared to fathers, although both would do the activity about once a month. Both expectant mothers and fathers almost never prayed to God (B_I_ = 1.25, SE = 0.45).

### Research Question 2: How does Frequency of Leisure Activities Change Over the Course of Pregnancy and Childbirth?

For mothers, from pregnancy week 19 to 1 year after childbirth, frequency of spending time just for herself (B_s_ = -0.46, SE = 0.19) and with friends (B_s_ = -0.27, SE = 0.14) decreased while frequency of praying to God increased (B_s_ = 0.17, SE = 0.07). For fathers, there were significant decreases in frequency of exercising (B_s_ = -0.49, SE = 0.20) over the course of pregnancy and after childbirth. Mothers did not change their frequency of listening to the news, exercising, or reading a newspaper or news tabloid and fathers did not change their frequency of listening to the news, reading a newspaper or news tabloid, spending time for self and with friends, or praying to God.

In other words, mothers and fathers converged for the activities where their leisure activity frequency differed from the other person (e.g., mothers more frequently spending time for herself and fathers more frequently exercising “dropped” to their partners’ level).

### Research Question 3: To What Extent is Frequency of Leisure Activities Associated Between Mothers and Fathers?

Around pregnancy week 19, all but spending time just for themselves had positive associations in frequency of activity between expectant mothers and fathers. In other words, the frequency of leisure during pregnancy was mirrored in partners for five of the six activities (Table 4). In couples where the mother spent more time on reading newspapers, the father also spent more time reading newspapers. This positive association also applied to listening to the news, exercising, spending time with friends, and praying. However, this was not the case for spending time for themselves. Mothers who spent more time for themselves are just as likely to have a partner who spends more time for themselves as a partner who spends less time for themselves. There is neither a positive nor a negative association.

Change over time was associated between couple members for one leisure activity (Table 4). Mothers’ change in listening to the news was associated with how much fathers changed in listening to the news. Thus, if mothers increased in how often they listened to the news, fathers are more likely to increase in their frequency, and vice versa. However, for the other activities, mothers’ and fathers’ change were not associated. How much mothers decreased in spending time with friends was not associated with fathers’ decrease in spending time with friends, and fathers’ decrease in exercise was not associated with mothers’ decrease in exercise.

## Discussion

How parents maintain their free time leisure activities can impact not only their own well-being, but also their caregiving capabilities. In this study, we examined how the frequency of leisure activities change during the transition to parenthood, a time of physiological, psychological, and social change (Bäckström et al., 2018; Klobučar, 2016). We find that expectant fathers were more frequently exercising and listening to the news, while expectant mothers spent more time reading newspapers or news tabloids, for themselves, and with friends. Over the course of pregnancy and childbirth, mothers spent less time for themselves and with friends, while increasing the frequency of praying, and fathers decreased how often they exercised. There were no changes in frequency for listening to the news or reading newspapers or news tabloids. Frequency of most leisure activities around pregnancy week 19 corresponded between the couples; however, most changes over time were not associated.

### Research Questions

#### How Does Frequency of Six Leisure Activities Differ Between Expectant Mothers and Fathers Around Pregnancy Week 19?

In our sample of expectant couples, we found gender gaps in listening to the news, exercising, reading newspapers and news tabloids, and spending time for self and with friends. Expectant fathers reported more frequent exercise, similar to previous findings on fathers (Wang, 2013). Since men do not need to physically deal with the physiological effects of pregnancy, including symptoms such as pain or nausea (Dekkers et al., 2020; Ray-Griffith et al., 2018), they may be more physically capable of exercising during pregnancy. This supports previous research, which finds that pregnancy-related symptoms are a barrier for physical activity (Coll et al., 2017). In addition, we found that fathers were also more likely to listen to the news on the radio, podcasts, or TV. Male partners of pregnant women maintain engagement in politics and societal issues during a pregnancy more so than pregnant women (Naurin et al., 2022), so expectant fathers may be more prone to interact with the news.

However, our results do not show that most differences would be to the advantage of the fathers. Expectant mothers reported more frequently reading a newspaper or news tabloid, spending time for themselves, and spending time with friends. Our finding that women read more text than men, combined with the finding that men listened more to the news, supports evidence that women read more than men (Bausch, 2014; Höglund & Wahlström, 2018; Ross et al., 2018), but men are more likely to read “through listening” (Asplund & Pérez Prieto, 2018; Tattersall Wallin & Nolin, 2020). Furthermore, we found that expectant mothers spend more time with friends, consistent with previous findings on mothers (Wang, 2013). Combined with a previous finding that men less frequently interact with family (i.e., parents, children) (Fingerman et al., 2020), it may be that men socialize less in general.

#### How does Frequency of Leisure Activities Change Over the Course of Pregnancy and Childbirth?

We found that frequency of leisure activities decreased during the transition to parenthood. However, gender differences in our sample decreased over time. From pregnancy to postpartum, mothers decreased how often they spent time for herself and with friends, narrowing the gender gap between mothers and fathers. This reduction in time spent for herself and with friends may reflect the new constraints to relaxation and fulfillment of personal needs (Kim et al., 2018) that occur after childbirth. Even though second trimester pregnancy (time point 1 in our study) may have limited her leisure, the birth of the child requires additional attention, which further reduces the amount of time she can spend by herself or with friends (Latshaw & Hale, 2016; Neilson & Stanfors, 2018). We found that mothers increased in praying to God, the only activity where the gender gap increased.

From around pregnancy week 19 to one year postpartum, fathers decreased the frequency of exercising, also narrowing the gender gap between mothers and fathers. We found that fathers exercised less over the course of pregnancy and early parenthood, similar to previous findings (Sjögren Forss & Stjernberg, 2019), which could be due to the increased responsibilities that the man has regarding housework and childcare (Eddy & Fife, 2021; Xue et al., 2018). Women’s exercise frequency remained stable, contrary to previous findings (Abbasi & van den Akker, 2015; Fazzi et al., 2017; Sjögren Forss & Stjernberg, 2019), but was on a lower level than men to begin with. Our finding that women do not decrease in exercise could be due to the fact that we started data collection during the second trimester. Unlike Abassi and van den Akker (2015) and Sjögren Forss and Stjernberg (2019), which examined activity levels prior to pregnancy, our data may have missed a drop in physical activity during the first trimester. However, these studies also found a decrease in activity during the pregnancy and after. Thus, the stability found in our data could also be due to several other reasons. First, women and care providers may have acted upon previous research and are actively seeking to prevent a decline in exercise during and after pregnancy. Second, it could be that the survey question lacks specificity. Since we only asked about exercise but not the type of exercise, it could be that our survey was not able to capture changes in exercise intensity (e.g., decreases in frequency of high-intensity workout but not walking or physiotherapy; Sjögren Forss and Stjernberg, 2019).

#### To What Extent is Frequency of Leisure Activities Associated Between Mothers and Fathers?

We found that the frequency of leisure activities of each couple member was associated during the second trimester of pregnancy. However, their changes were not associated over time. Frequency of listening to the news, exercising, reading a newspaper, spending time with friends, and praying to God around pregnancy week 19 was associated between expectant couple members. This may be due to partner selection mechanisms, where individuals who have similar characteristics often become couples (Luo, 2017). More involved, active, and social mothers may have partners who are also more involved, active, and social. However, mothers’ spending more time for herself was not significantly associated with fathers’ spending time for himself. While this may suggest that spending time for oneself is not a couple-dependent activity, it could also be that at the beginning of pregnancy, the couple members were not on parental leave yet, and that the time spent for themselves is more dependent on their careers and personal life outside of the pregnancy rather than their partner. Alternatively, as mentioned previously, the lack of association may also be due to men and women interpreting this activity question differently (Woolhouse et al., 2016).

In general, aside from listening to the news, change in leisure activity participation was not associated between couple members throughout pregnancy and childbirth. Thus, while couple members may influence each other’s behaviors (Abbass-Dick et al., 2015; Umberson et al., 2018), this finding suggests that during the transition to parenthood, other factors (e.g., pregnancy effects, child’s temperament) outside of the partner may influence these leisure activity changes.

### Limitations and Future Research Directions

There were a few limitations associated with this study. First, our data was limited in the sense that frequency of common leisure activities, such as reading for pleasure and watching television, were not captured. Second, participants self-reported on how often they spent time on individual leisure activities in the past three months. Thus, the measure of leisure may not have been sensitive enough to capture change in time spent on the activities. Responses to this question may include leisure at points prior to when the survey was sent out or time simultaneously spent alongside another activity (i.e., not solely time spent on one leisure activity). However, by asking participants to generalize their leisure over a certain period, we avoid the risk that they think about how their life normally is (i.e., long before the pregnancy). Furthermore, in our study, since we ask about specific leisure activities rather than have participants define their own leisure (see Kamp Dush et al., 2018), we reduce the risk of finding spurious differences, where different people define leisure time differently.

Third, our data was collected in 2019–2021, during the COVID-19 pandemic, which could have disrupted leisure time for couples. However, Sweden’s liberal response to the pandemic, such as keeping society open (Krisinformation, 2020; Pashakhanlou, 2021), may have prevented disruption of some leisure activities for both expectant mothers and fathers. However, the general disruption caused by COVID-19, along with additional health concerns, could have impacted individual parent leisure trajectories, and how expectant couples influenced each other, similar to how COVID-19 impacted relationship quality amongst couples (Pietromonaco & Overall, 2021). In addition, we recognize that certain activities in our study, such as reading or listening to the news, may have become less of a leisure activity during the COVID-19 pandemic. Beyond the worry these activities may spark, excessive leisure of any kind, whether obsessive reading of the news or extended periods of spending time alone, can trigger a downward shift in well-being.

Fourth, our data was collected in one context. Previous studies have found that leisure gender gaps in Sweden are small compared to other Western countries (Anxo et al., 2011), indicating that our findings capture this transition in an otherwise gender-equal context when it comes to leisure. In addition, even though our sample is large (918 couples), it is skewed towards participants who are more highly educated, less religious, and living in a setting with extensive public support, which might affect the generalizability of the findings. Similarly, participants who dropped out of the study were less educated and younger, which might limit the generalizability of the observed trajectories. However, this was a highly appropriate sample to test the extent to which leisure activities may be similar or different between men and women in a context where there are reduced gender norm constraints on women (Duvander et al., 2005; Ferrant et al., 2014; Miller & Brown, 2005; Oláh & Bernhardt, 2008; Thomsson, 1999). Furthermore, by using a couples-only sample, our findings may reflect leisure frequency and changes that occur in couples who share similar interest in the pregnancy or availability to complete the survey. Our findings may be less generalizable to couples where one partner does not respond due to lack of time, interest, or investment in the pregnancy.

Finally, we note several analytical limitations. In our analyses, we control for two variables: education and income. That is due to the fact that Sweden has extensive public support for individuals and families (free comprehensive routine care for all pregnant women, free parental education and support for partners, generous parental leave) which aims at evening out differences between citizens. Thus, to prevent over-controlling of the data, which can lead to spurious associations, we only control for these two factors that do vary in our data. There may still be factors affecting how time is spent, such as work time and time spent on housework, even within the relatively homogenous Swedish sample. However, in our sample we do not have another variable directly measuring how people spend time. Future research can highlight how frequency of leisure may be influenced by time spent on other activities, such as work or household chores, as well as how that frequency may vary within couples themselves. Finally, expectant parents’ time for self could not be captured fully by a linear, quadratic, or piecewise latent growth curve. This suggests that for this leisure activity, there may be oscillations in trajectory over time due to different physiological responses or demands on one’s time that cannot be captured consistently across time (Dekkers et al., 2020; Ray-Griffith et al., 2018; Sel, 2020).

Despite these limitations, this study offers a novel perspective into gender differences in mothers’ and fathers’ frequency of leisure activities during the transition to parenthood. Combined with previous research, these findings posit the need for research to clarify how changes in parent activities impact not just parent but also child well-being, as well as the different types of leisure activities and contexts that may contribute to gender gaps in leisure. Further research may need to clarify the relationships of individual leisure activities over time, from before the onset of pregnancy to later parenthood, since changes may have taken place prior to the second trimester of pregnancy. In addition, a next step is to more closely investigate the consequences of pregnancy and childbirth for fathers, as a focus on mothers may underestimate the effects on the father. Finally, more studies are needed to understand the interplay between frequency and the quality of leisure time, since the benefits obtained from leisure time may be impacted by the degree of relaxation and enjoyment.

### Practice Implications

Leisure is linked to emotional and physical health (Demirbaş, 2020; Moses et al., 2016; Paggi et al., 2016; Qian et al., 2014; Schmiedeberg & Schröder, 2017; Zurawik, 2020) and improved attachment with the unborn child (Chang et al., 2015; Nivedhitha & Geetha, 2022). In our study, this convergence in leisure routines could indicate that generally, gender equality and health can be maintained, even during this time of physiological and social change. While this comes at a cost for both the mother and father (i.e., both decrease their frequency of leisure activities), at the same time, it makes sense that leisure time is redistributed when a child enters one’s life. Our finding that the transition to parenthood may not exacerbate gender differences brings hope that in certain contexts, parenthood may not create further disparities in childcare and parenting outcomes. However, following from this, it is possible that past research during this life transition, by focusing on mothers alone, has underestimated effects on the father. As a result, less may be known about the consequences of pregnancy and childbirth for fathers, including but not limited to changes in attitudes (Buchler et al., 2017) and parental burnout (Roskam & Mikolajczak, 2020), and whether fathers who do experience these changes “bounce back” quicker than mothers (Bhatti et al., 2019; Naurin et al., 2022).

## Conclusion

Frequency of leisure activities represents an important component of parental self-development and well-being, as these activities reflect one’s needs, values, and goals. During the transition to parenthood, mothers and fathers may find that competing expectations (personal versus child well-being) alter their leisure routines and role as an individual within the family unit. However, this study suggests that while there are changes in leisure during this important time, the changes are not limited to only one parent or one type of leisure activity. Thus, in this study, conducted in a context with state support for family and gender egalitarian norms, we find that the transition to parenthood is a balancing act between mothers and fathers. Mothers and fathers both adjust their frequency of leisure during late pregnancy and first year of parenthood and narrow some gender gaps in leisure activities.

This is the first study to use longitudinal dyadic data on couples, analyze frequency of individual leisure activities, and examine these associations from pregnancy to parenthood. This study also offers a novel look into how time spent outside of housework and childcare may also contribute to the gender differences between heterosexual couples seen in parenthood. Understanding well-being in the context of couplehood and parenthood poses an alternative perspective for understanding the health of the child and family, since “the kids will be alright if the adults who care for them are, too” (Racine et al., 2020, p. 508).

## Data Availability

The datasets generated during and/or analyzed during the current study are not publicly available due to the nature of the survey and register data (health information and sensitive personal behavior).

## Code Availability

The code generated during and/or analyzed during the current study are available from the corresponding author on reasonable request.
